# Flaw Detection in White Porcelain Wine Bottles Based on Improved YOLOv4 Algorithm

**DOI:** 10.3389/fbioe.2022.928900

**Published:** 2022-07-11

**Authors:** Guoqiang Gong, Jun Huang, Hemin Wang

**Affiliations:** College of Computer and Information Technology, China Three Gorges University, Yichang, China

**Keywords:** flaw detection, white porcelain bottle, YOLOv4, CA mechanism, deformable convolution, loss function

## Abstract

Aiming at the problems of low detection accuracy and slow detection speed in white porcelain wine bottle flaw detection, an improved flaw detection algorithm based on YOLOv4 was proposed. By adding Coordinate Attention to the backbone feature extraction network, the extracting ability of white porcelain bottle flaw features was improved. Deformable convolution is added to locate flaws more accurately, so as to improve the detection accuracy of flaws by the model. Efficient Intersection over Union was used to replace Complete Intersection over Union in YOLOv4 to improve the loss function and improve the model detection speed and accuracy. Experimental results on the surface flaw data set of white porcelain wine bottles show that the proposed algorithm can effectively detect white porcelain wine bottle flaws, the mean Average Precision of the model can reach 92.56%, and the detection speed can reach 37.17 frames/s.

## Introduction

With the rapid development of social economy, the liquor industry in China is also developing rapidly. In order to ensure the sustainable development of enterprises, every liquor production enterprise needs to strictly control the quality of liquor products. As the most popular container for most liquor products, the quality of white porcelain bottles directly affects the quality of liquor products, and thus white porcelain bottle flaw detection is very important.

At present, there are mainly two kinds of white porcelain wine bottle flaw detection techniques: (1) manual detection and (2) machine vision detection. Traditional manual detection is greatly influenced by subjectivity and has problems in terms of low detection accuracy and low detection efficiency. With the development of image processing technology, machine vision technology began to be applied to white porcelain bottle flaw detection. For the quality detection of empty bottles, the In-Line empty bottle detection machine developed by Haifu Company has a good detection effect (HEUFT SYSTEM TECHNIK, 2022a). For full bottle quality detection, the PRIME detector developed by Haifu can realize liquid level detection of wine bottles by using special sensors and optical technology (HEUFT SYSTEM TECHNIK, 2022b). For the quality inspection of printed matter on the surface of wine bottles, the FA-Falcon Automatic inspection system, the latest product of Israel Avit Company, can detect the flaws of printed matter on wine bottles (Li, 2020). Although the existing machine vision inspection technology has been achieved, the existing bottle flaw detection technology of detecting speed is slow, where the fastest can only reach 75,000 bottles per hour; however, the current beverage line production demand has reached 100,000 bottles per hour, in which traditional machine vision inspection technology has been unable to meet. In addition, the traditional machine vision detection technology still has some problems, such as time-consuming manual design and single detection algorithm function. Therefore, it is of great significance to develop an intelligent and efficient white porcelain wine bottle flaw detection method.

At present, there are many widely used models of deep learning, which are mainly divided into two categories: 1) one-stage algorithm represented by SSD, YOLO, YOLOv2, YOLOv3, YOLOv4, YOLOv5, and other networks (Liu et al., 2016; Redmon et al., 2016; Redmon and Farhadi, 2017; Redmon and Farhadi, 2018; Bochkovskiy et al., 2020); 2) The other is a two-stage algorithm represented by R-CNN, Fast R-CNN, Faster R-CNN, Mask R-CNN, and other networks (Girshick et al., 2014; Girshick, 2015; He et al., 2017; Ren et al., 2017). The advantage of a two-stage algorithm is that it has high detection accuracy. However, due to its complex network structure, the detection speed of the algorithm is slow. For example, Du et al. implemented semantic segmentation, target classification, and multi-visual task detection in indoor scenes by using the improved Faster-RCNN algorithm (Jiang et al., 2021). Gao et al. (2019) proposed a tunnel flaw detection method based on improved Faster R-CNN, which has higher accuracy compared with traditional algorithms. He et al. (2019) proposed a flaw detection algorithm for steel plates based on Faster R-CNN, and the average accuracy of flaw detection reached 82%. Tao et al. (2018) designed a cascade network based on Faster R-CNN for insulator flaw detection. After testing the insulator data set, the average accuracy of flaw detection reached 91%, but the detection time of a single image was 360 ms, and the detection speed was relatively slow. On the contrary, the one-stage algorithm has the advantage of fast detection speed, but low detection accuracy. For example, many researchers have applied the one-stage target detection algorithm to indoor small target detection, medical image detection, industrial safety production, and industrial quality inspection (Huang et al., 2021; Tsai et al., 2021; Huang et al., 2022). Wei et al. (2020) proposed a railway track flaw identification method based on image processing and improved YOLOv3, and the detection speed reached 33 frames/s. Liao et al. (2021) proposed an algorithm for PCB surface flaw detection based on improved YOLOv4, which achieved a detection speed of 56.98 frames/s without detection and improved detection accuracy. Qiu et al. (2022) proposed an improved YOLOv4-tiny algorithm for flaw detection of wood panels. Res2Net was used as the backbone feature extraction network, resulting in average detection accuracy of the algorithm up to 80.1%, and the detection speed up to 76.9 frames/s. Liu Q. et al. (2022) proposed an improved YOLOv4 algorithm for fabric flaw detection. A new SPP structure was adopted and SoftPool was used instead of MaxPool, which made the average accuracy of fabric flaw detection achieve 86.5%. Liu X. M. et al. (2022) proposed an improved YOLOv4 algorithm for insulator flaw detection and improved the convolutional layer of the trunk feature extraction network, resulting in the average detection accuracy of the algorithm up to 84.05%, with a detection speed up to 30.6 frames/s. Sun et al. (2022) replaced CSPDarknet53, the backbone feature extraction network of YOLOv4, with an improved MobileNetV3 network for stamping flaw detection, which improved the detection speed by 4 frames/s but reduced the detection accuracy. Yang and Sang (2022) proposed a multi-scale feature adaptive fusion lightweight fabric flaw detection algorithm, using MobileNetv2 as the main feature extraction network of YOLOv4, with the addition of the Coordinate Attention module, improving the average detection accuracy of the algorithm by 2.3%. The detection speed reached 26 frames/s. The aforementioned literature fully shows that the current bionics algorithm based on deep learning has good learning ability and can meet the needs of some fields. Therefore, the deep learning-based bionics algorithm is adopted in this research for white porcelain wine bottle flaw detection. This study is the specific application of the bionics algorithm in the quality inspection of white porcelain wine bottles, which is of great significance for white porcelain wine bottle bionics detection. At the same time, this research can promote the development of bionics and biomimetics and has a certain reference significance for the research and application of bionics algorithms in other industrial quality inspections. In addition, it can be seen from the above literature that YOLO series algorithms can be applied to flaw detection. Such algorithms have the characteristics of fast detection speed, but the detection accuracy of the original YOLO series algorithms is not high and needs to be improved for specific problems.

With the continuous development of YOLO series algorithms, their shortcomings are gradually improved, and the current YOLOv4 algorithm has better detection speed and accuracy. Although the YOLOv5 algorithm model is small, the detection accuracy of the algorithm is low especially for small targets, the flaw detection effect is poor and thus unsuitable for the high precision requirements of white porcelain bottle flaw detection.

Therefore, in order to improve the detection performance of white porcelain wine bottle flaws, this research adopts the improved YOLOv4 algorithm. First, images of different types of white porcelain bottle flaws were collected to construct data sets. Second, CA (Coordinate Attention) was added to the feature extraction network of YOLOv4 to improve the extraction ability of the white porcelain bottle flaw detection model. At the same time, deformable convolution is added to locate flaws of different shapes and sizes more accurately, so as to improve the detection accuracy of flaws by the model. In addition, the loss function was improved and CIoU in YOLOv4 was replaced by EIoU to improve the model detection accuracy and detection speed. The white porcelain wine bottle flaw detection algorithm proposed in this research effectively improves the detection accuracy and has a fast detection speed, thus satisfying the real-time requirements of white porcelain wine bottle flaw detection in industrial settings.

## YOLOv4 Algorithm

YOLOv4 algorithm is improved on the basis of the YOLOv3 algorithm by appropriately integrating the innovative points of various advanced algorithms. It is the algorithm with the highest detection accuracy in YOLO series algorithms. The network structure the of YOLOv4 algorithm consists of three parts: backbone network, neck, and head. The backbone is mainly responsible for feature extraction, the neck is mainly responsible for feature fusion, and the head is mainly responsible for detection.

The backbone of YOLOv4 uses CSPDarknet53 as the backbone feature extraction network. CSPDarknet53 is a combination of the multi-channel network (CSP) and Darknet53 (Redmon and Farhadi, 2018; Wang et al., 2020) and uses a faster Mish activation function. The neck part adopts FPN + PANet structure to carry out feature aggregation for different detection layers from different trunk layers, so as to have a strong feature extraction ability (Lin et al., 2017; Liu et al., 2018). In addition, the SPP module is also adopted to increase the receiving range of trunk features and separate significant context features more effectively (He et al., 2015). The head part still adopts YOLOv3 detection head, but the loss function part of the target detection task is improved. CIoU Loss is used as a regression loss function to improve detection speed and accuracy, and DIoU NMS is used to screen prediction boxes to improve the detection accuracy of overlapping targets (Zheng et al., 2020).

In addition, YOLOv4 also adopts a series of methods such as Mosaic data enhancement, CmBN, and SAT self-adversarial training to optimize the algorithm (Bochkovskiy et al., 2020). Among them, Mosaic data enhancement greatly enriches the detection data set and reduces the GPU overhead.

As the algorithm with the highest detection accuracy in the YOLO series, YOLOv4 has a strong learning ability and can be applied to flaw detection of some products. Flaw detection using YOLOv4 is mainly divided into two steps. Step one is the training network model: the YOLOv4 algorithm uses the data set to train, according to the loss function for several times of reverse iteration, constantly update the network parameters, make the network more and more accurate, and finally trained into a network model. Step two is the model test, which uses the trained network model to test the input image, and finally completes the detection and positioning of flaws in the image.

## Improved YOLOv4 Algorithm

**FIGURE 1:**
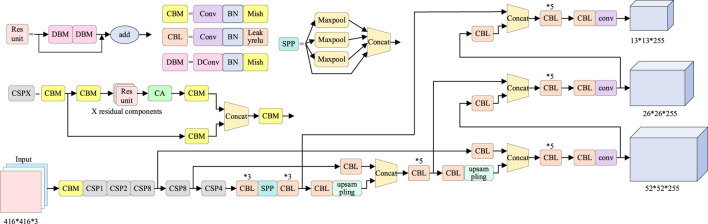
Network structure of the improved YOLOv4 algorithm.

The algorithm proposed in this research is improved on the basis of YOLOv4, which mainly includes three aspects. First, the CA module of the attention mechanism is added to the CSP module of the CSPDarkNet53 backbone feature extraction network of YOLOv4. Second, all 3 × 3 convolution in CSPDarknet53 residual block of YOLOv4 feature extraction network is changed to deformable convolution. Third, change the loss function DIoU in YOLOv4 to EIoU. The network structure diagram of the improved algorithm is shown in Figure 1.

### CA Mechanism

The attention mechanism is very important for neural network, and the addition of an attention mechanism can improve the network’s attention to important feature information and reduce the attention to irrelevant information. In the collected white porcelain bottle image, the flaw occupies only a small part of the image, and most of the image is background information. In the training process, information redundancy will be generated when a large number of background information is iterated, leading to partial flaw target information being submerged, thus affecting the detection accuracy. In addition, many flaws in the white porcelain wine bottle image are small target flaws, and the original YOLOv4 algorithm has poor extraction ability for small targets, so it is difficult to detect these small target flaws. After the attention mechanism is added, the attention of the network to flaws is increased, and at the same time, the network will notice these small target flaws, so as to improve the accuracy of small target detection of the network.

**FIGURE 2:**
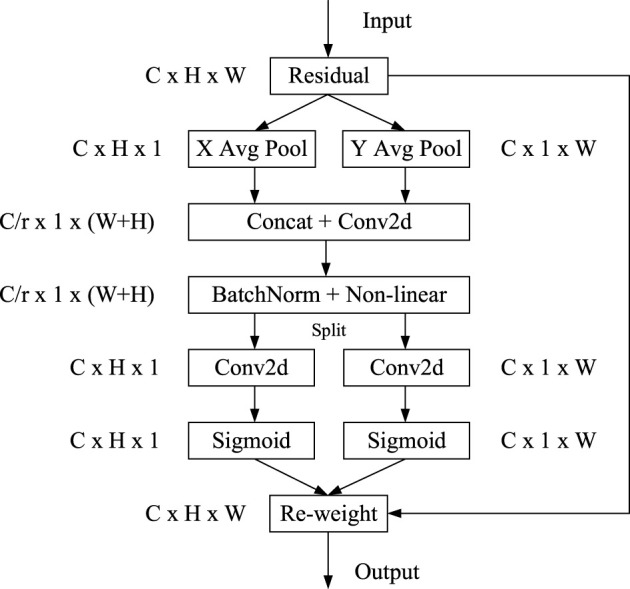
CA module structure.

There are a number of attention mechanisms available, for example, SE (Squeeze and Congestion), CBAM (Convolutional Block Attention Module), and CA (Coordinate Attention). (Hu et al., 2018; Woo et al., 2018; Hou et al., 2021). The CA mechanism is a lightweight mobile network that empowers location information into the attention channel. The attention mechanism can obtain not only cross-channel information but also direction perception and position perception information, so that the model can locate and identify the flaw target more accurately. Therefore, in this research, the CA mechanism was added to the YOLOv4 network structure to improve the attention channel of the network to the flaw target, so as to improve the accuracy of flaw detection. The structure of the CA mechanism is shown in Figure 2.

CA mainly includes two steps: the first step is coordinate information embedding. Given an input: $F\in R^{H*W*C}$ , the global mean pooling is divided into a pair of one-dimensional pooling, that is, two pooling cores (H, 1) and (1, W) are pooled along the horizontal and vertical directions of the feature graph. Two embedded information feature graphs $Z_{c}^{h}$ and $Z_{c}^{w}$ are obtained, and the output formula is given as Eqs. 1 and 2:

$$Z_{c}^{h}\left(h\right)=\frac{1}{W}\underset{0\leq i\leq W}{\sum }x_{c}\left(h,i\right)\;\;\;\;Z_{c}^{h}\in R^{C*H*1},\;\;$$ (1)

$$Z_{c}^{w}\left(w\right)=\frac{1}{H}\underset{0\leq j\leq H}{\sum }x_{c}\left(j,w\right)\;\;\;Z_{c}^{w}\in R^{C*1*W}.$$ (2)

The second step is the generation of a coordinate information feature map. First, the two obtained embedded feature graphs $Z_{c}^{h}$ and $Z_{c}^{w}$ W are splicing along the spatial dimension. After 1*1 convolution function $F_{1}$ transformation, the nonlinear activation function δ is used to activate and generate process feature graph F, as shown in formula 3:

$$f=\delta \left(F_{1}\left(\left[z^{h},z^{w}\right]\right)\right).$$ (3)

Second, two separate feature graphs $f_{h}$ and $f_{w}$ were obtained by Split operation along the spatial dimension. After the 1*1 convolution function $F_{h}$ and $F_{w}$ were transformed respectively, the attention vectors $g^{h}$ and $g^{w}$ were obtained by Sigmoid activation function σ activation, as shown in formulas 4 and 5:

$$g^{h}=\sigma \left(F_{h}\left(f_{h}\right)\right),$$ (4)

$$g^{w}=\sigma \left(F_{w}\left(f_{w}\right)\right).$$ (5)

Finally, $g^{h}$ and $g^{w}$ are extended to be multiplied by the input feature graph as the attention weight value to obtain the final feature graph of attention. The output formula is as Formula 6:

$$y_{c}\left(i.j\right)=x_{c}\left(i.j\right)\times g_{c}^{h}\left(i\right)\times g_{c}^{w}\left(j\right).$$ (6)

**FIGURE 3:**
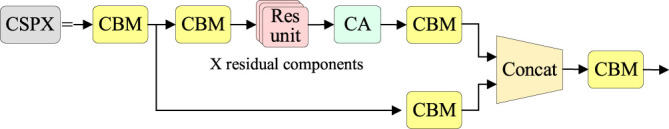
Improved CSP module.

In order to highlight the white porcelain wine bottle flaw features, accurately locate and identify flaws, and improve the accuracy of white porcelain wine bottle flaw detection, this research added the CA module of attention mechanism into the CSP module of CSPDarkNet53 backbone feature extraction network of YOLOv4 and took it as a discriminant feature filter. Feature information of detection targets can be extracted more effectively to improve detection accuracy. The improved CSP module is shown in Figure 3.

### Deformable Convolution Networks

**FIGURE 4:**
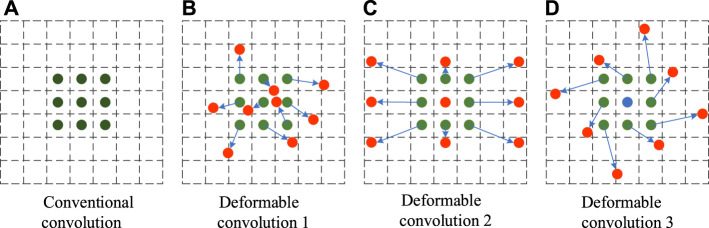
Comparison of conventional convolution and deformable convolution sampling points. **(A)** is the sample point graph of the conventional convolution. **(B–D)** are sample point graphs of the deformable convolution.

The conventional convolution used in the YOLOv4 model can only sample-fix positions in the feature graph, and this convolution kernel can extract rectangular features well. However, the white porcelain wine bottle flaws are characterized by various forms, and the white porcelain flaws cannot be well located by conventional convolution, thus affecting the accuracy of the flaw detection model. However, deforming convolution adds a training factor that can be changed to the conventional convolution module, so that the size and position of the convolution kernel can be dynamically adjusted according to the characteristics of the input image, and the position of the convolution kernel sampling points at different positions will change adaptively according to the characteristics of the image (Dai et al., 2017). Therefore, deformable convolution can locate flaws of different shapes and sizes more accurately. The sampling point pairs of conventional convolution and deformable convolution are shown in Figure 4.

As can be seen from the figure above, conventional convolution can only carry out regular sampling, while deformable convolution can carry out random sampling around the current position, making the sampling position more suitable for the shape and size of flaws and thus better feature extraction.

The calculation process of deformable convolution is improved on the basis of conventional convolution, which mainly consists of two steps. The first step is to use regular grid R to sample the feature graph X. The second step is the sum of each sample point multiplied by the weight. The output formula is given as Formula 7:

$$y\left(p_{0}\right)=\underset{p_{n}\in R}{\sum }w\left(p_{n}\right)\cdot x\left(p_{0}+p_{n}\right)\;.$$ (7)

In the aforementioned formula, $p_{n}$ is the enumeration of each position in grid R, and $w\left(p_{n}\right)$ is the weight of the corresponding point. The deformable convolution is to expand the regular grid R by adding an offset { $\Delta p_{n}|n=1,\ldots ,N\},N=|R|$ . Its output formula is shown in Formula 8 .

$$y\left(p_{0}\right)=\underset{p_{n}\in R}{\sum }w\left(p_{n}\right)\cdot x\left(p_{0}+p_{n}+\Delta p_{n}\right).$$ (8)

Since $\Delta p_{n}$ is usually a decimal, the bilinear interpolation method is required to calculate the value of x(p) , as shown in Formula 9.

$$x\left(p\right)=\underset{q}{\sum }G\left(q,p\right)\cdot x\left(q\right).$$ (9)

**FIGURE 5:**
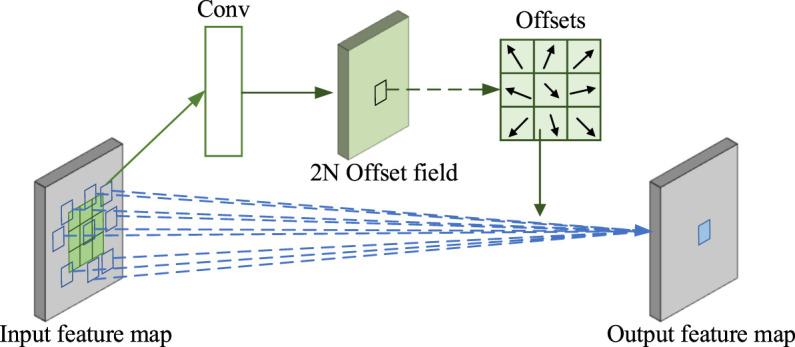
Schematic diagram of deformable convolution.

In the aforementioned formula, $p=p_{0}+p_{n}+\Delta p_{n}$ , *q* is the enumeration of *x* spatial position of the feature graph, and *G(,)* is the bilinear interpolation function. The feature extraction process of deformable convolution is shown in Figure 5.

As can be seen from the diagram shown earlier, deformable convolution in the conventional convolution joined in a convolution layer is used to calculate the offset. Moreover, because the offset calculation and output characteristics of convolution kernels are parallel, the size and location of deformable convolution kernels can dynamically adjust according to the characteristics of the input feature maps, and thereby learn different forms of white porcelain bottle flaws.

**FIGURE 6:**
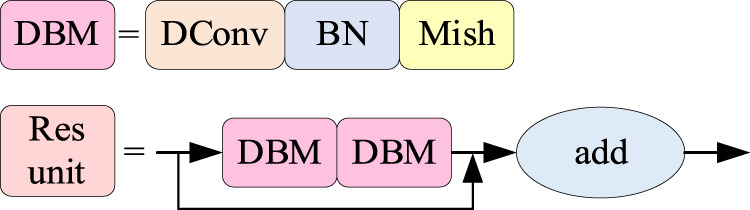
Residual block network structure with deformable convolution.

Deformable convolution can improve the modeling ability of the model for flaws of different shapes and sizes, and thus improve the accuracy of flaw detection. In this research, all 3 × 3 convolution in CSPDarknet53 residual block of YOLOv4 feature extraction network is changed to deformable convolution to form a new and more powerful feature extraction network. The structure of the modified residual block network is shown in Figure 6.

### Optimized Loss Function

The calculation of the loss function can judge whether the parameters of the current training model meet the standard and reflect the difference between the training model and the real data (Hao et al., 2022). Therefore, the loss function is very important for the training of the model. Selecting an appropriate loss function can train a model with a better detection effect and improve the convergence speed of model training.

The loss function of YOLOv4 consists of three parts, namely: classification loss, position loss, and confidence loss. The calculation of position loss is mainly to determine the position of the detection target. In YOLOv4, the CIoU Loss function is used to calculate the regression Loss of the target box, and its calculation formula is shown in Eqs 10–13:

$$L_{CIoU}=1-IoU+\frac{\rho^{2}\left(b,b^{gt}\right)}{C^{2}}+\alpha v,$$ (10)

$$IoU=\frac{\left|B\cap B^{gt}\right|}{\left|B\cup B^{gt}\right|},$$ (11)

$$v=\frac{4}{\pi^{2}}{\left(arctan\frac{w^{gt}}{h^{gt}}-arctan\frac{w}{h}\right)}^{2},$$ (12)

$$\alpha =\frac{v}{\left(1-IoU\right)+v}.$$ (13)

In the aforementioned formulas, *B* is the size of the prediction frame; $B^{gt}$ is the size of the real frame; IoU is the ratio of the intersection and union between the prediction frame and the real frame; *b* and $b^{gt}$ , respectively, represent the center point of the prediction frame and the real frame; *ρ(,)* represents the Euclide distance between the two points; *C* is the diagonal distance of the smallest rectangle containing the prediction frame and the real frame; $w^{gt}$ and $h^{gt}$ represent the width and height of the real box, respectively; and *w* and *h* represent the width and height of the prediction box, respectively.

A good regression function of the target box needs to include three important factors, such as the overlap area between the prediction box and the real box, the distance between the center point, and the aspect ratio, which are taken into account by the CIoU Loss function. However, in CIoU Loss, only *v* reflects the difference in aspect ratio but does not reflect the real relationship between the aspect ratio of the prediction frame and the real frame. In this way, CIoU Loss may unreasonably optimize the similarity. Therefore, EIoU Loss was adopted in this research to replace CIoU Loss to calculate the target box regression Loss. EioU Loss function improves its aspect ratio on the basis of the CioU Loss function (Zhang et al., 2021). EioU Loss calculates the length and width of the prediction frame and target frame separately, and its calculation formula is shown in Formulas 14 and 15.

$$L_{EIoU}=1-IoU+\frac{\rho^{2}\left(b,b^{gt}\right)}{C^{2}}+L_{asp},$$ (14)

$$L_{asp}=\frac{\rho^{2}\left(w,w^{gt}\right)}{C_{w}^{2}}+\frac{\rho^{2}\left(h,h^{gt}\right)}{C_{h}^{2}}.$$ (15)

In the aforementioned formula, $C_{w}$ and $C_{h}$ are the width and height of the minimum rectangle containing the prediction box and the real box, respectively. $L_{asp}$ indicates the aspect ratio Loss of the EIoU Loss function.

EIoU Loss not only retains the advantages of CIoU Loss but also directly minimizes the width and height differences between the target frame and the prediction frame, thus accelerating the convergence speed and achieving better target positioning results.

## Experiment

### Experimental Environment

The operating system used for the research presented in this research is CentOS Linux 7, the graphics card is NVIDIA GEFORCE RTX 1080Ti, and the video memory is 12 GB. In this research, the deep learning framework of PyTorch is used for experiments. The experimental environment is Python 3.6 and CUDA 10.1.

### Experimental Data Set

**FIGURE 7:**
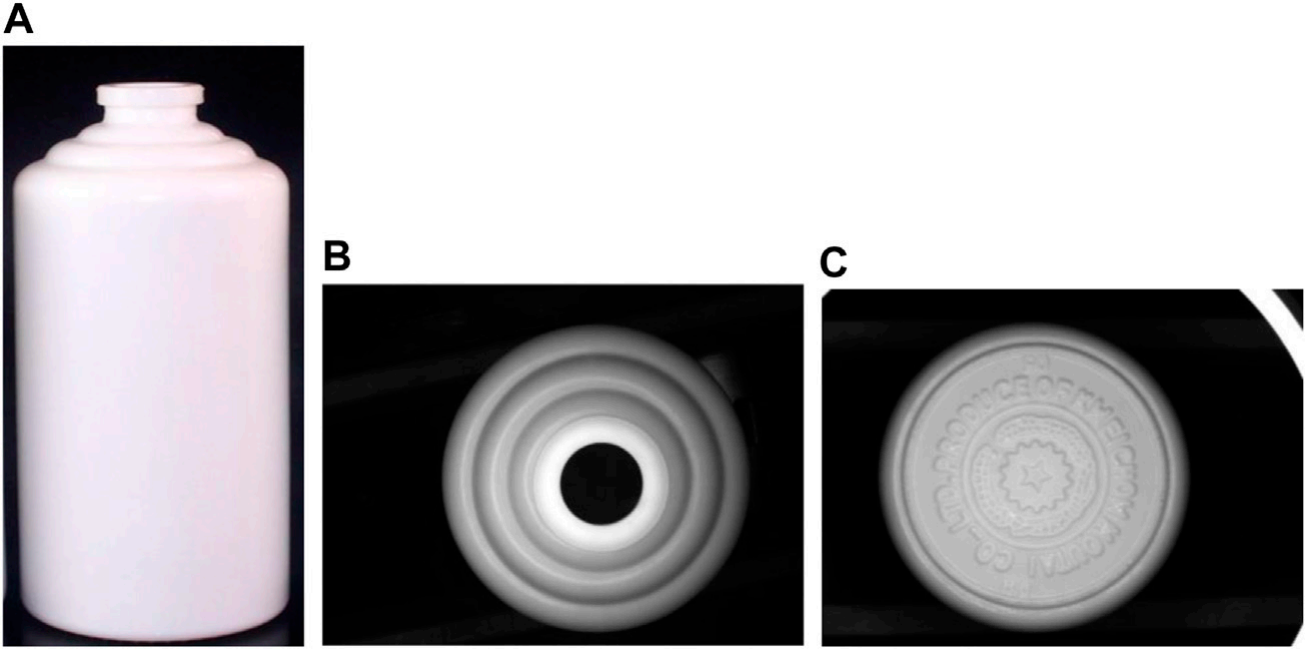
Overall picture of a white porcelain bottle. **(A)** is the side view of the white porcelain bottle, **(B)** is the top view of the bottle mouth, **(C)** is the bottom view of the white porcelain bottle.

In this research, white porcelain bottles of the same form and type were used for the experiment. The shape of the white porcelain bottle used in the experiment is shown in Figure 7. As can be seen from Figure 7, the white porcelain bottle has a flat mouth. From the top of the bottle, the shape of the bottle mouth is round and circular. The shoulder part of the white porcelain bottle is three layers of smooth rings. The body part of the white porcelain bottle is a smooth cylinder. The bottom part of the white porcelain bottle has raised rings, patterns, and words.

**FIGURE 8:**
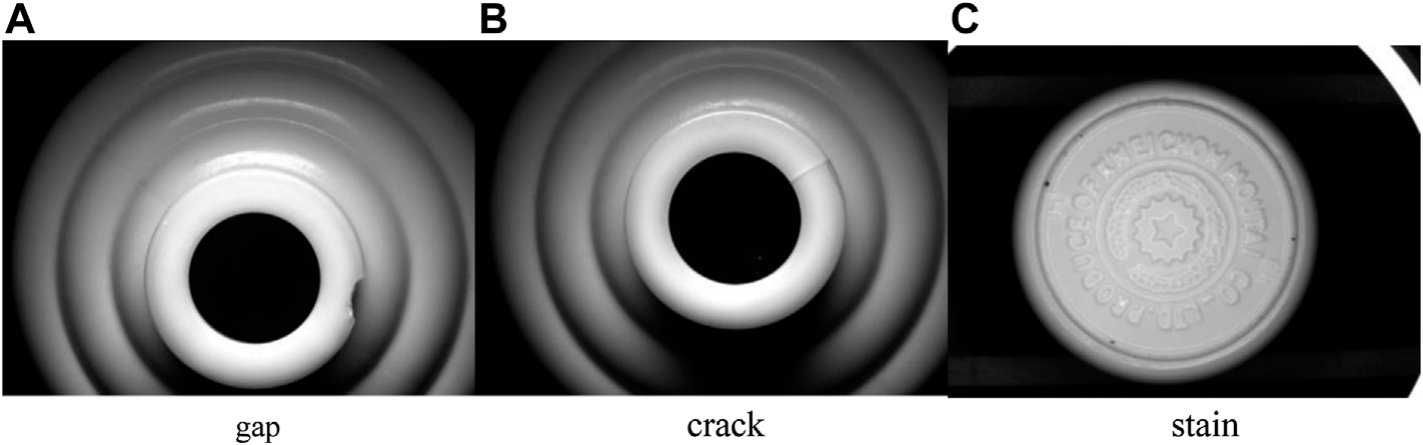
Three flaws in a white porcelain wine bottle. **(A)** is the picture of the gap of the white porcelain bottle, **(B)** is the picture of the crack of the white porcelain bottle, **(C)** is the picture of the stain of the white porcelain bottle.

The data set photos used in this research are the surface flaw pictures of white porcelain wine bottles collected from the production line of a wine enterprise. The collected photos mainly feature the bottle mouth and bottle bottom, and the collected images include notches, cracks, and stains, as shown in Figure 8.

In this research, the LabelImg tool was used to mark the flaws in the images, and the data set of this research was made in accordance with the format of VOC 2007, and the sample number of the final data set was 6,000.

### Evaluation Index

In order to verify the performance of the improved YOLOv4 algorithm, this research adopted the mean average precision (mAP) and detection speed FPS (frames per second) of multiple categories as evaluation indexes.

Average precision (AP) refers to the average accuracy of a single type of detection, which is used to measure the detection accuracy of an algorithm for a single type. The mean average precision (mAP) refers to the average value of average accuracy AP of multiple categories, which is used to measure the detection accuracy of the algorithm for all categories. Its calculation formula is shown in Formulas 16 and 17.

$$AP=\overset{1}{\underset{0}{\int }}P\left(R\right),$$ (16)

$$mAP=\frac{1}{c}\overset{c}{\underset{j=1}{\sum }}AP_{j}.$$ (17)

In the aforementioned formula, *P* is precision, *R* is recall, and *P(R)* represents the accuracy–recall curve. The calculation formulas of accuracy *P* and recall rate *R* are shown as Formulas 18 and 19.

$$Recall=\frac{TP}{TP+FN},$$ (18)

$$Precision=\frac{TP}{TP+FP}.$$ (19)

In the aforementioned formulas, *TP* is the true sample, indicating the sample whose detection result is the same as the actual result; *FN* is a false negative sample, indicating no actual sample detected; and *FP* is a false sample, which means that the test results are different from the actual results.

FPS (frames per second) refers to the number of photos that can be detected by the algorithm model per second, which is used to measure the detection speed of the algorithm for the target.

### Model Training

In this research, 6,000 photos of white porcelain wine bottles were marked for flaws and randomly divided into training sets and test sets in a ratio of 9:1, of which 5,400 were in the training set and 600 in the test set. In this research, the input images were enhanced with Mosaic data for training. A total of 300 batches were trained. The batchsize of the first 150 batches was set as 64 and the learning rate was set as 0.001, while the batchsize of the last 150 batches was set as eight and the learning rate was set as 0.0001. Num_workers was set to 4. A total of five models were trained in the experiment in this research, namely, Faster-RCNN, YOLOv3, YOLOv4, YOLOv5, and the improved YOLOv4 model in this research.

### Contrast Experiment

In order to verify the effectiveness of the improved algorithm, several typical target detection algorithms are selected for comparative experiments. In this research, the Faster-RCNN, YOLOv3, YOLOv4, and YOLOv5 models were, respectively, used for training and verification on the white China wine bottle data set. The comparison results of AP, mAP, and FPS of each model are shown in Table 1. In addition, in order to better prove the performance of the improved algorithm, the experimental results in this research are compared with the results of the latest YOLOv4 flaw detection research, as shown in Table 1.

**TABLE 1:** Comparison of detection performance of different algorithms.

Model	mAP/%	FPS/(frames/s)
Faster-RCNN	83.32	4
YOLOv3	81.23	25.4
YOLOv4	87.36	38.5
YOLOv5	85.18	44.32
Qiu et al. (2022)	80.1	76.9
Yang and Sang (2022)	91.67	26
Improved YOLOv4	92.56	37.17

In Table 1, Qiu et al. (2022) and Yang and Sang (2022) are the two latest research studies selected for flaw detection using YOLOv4, among which (Qiu et al., 2022) uses the improved YOLOV4-tiny algorithm for wood panel flaw detection. Flaw detection using the YOLOV4-tiny algorithm greatly improves the detection speed. At the same time, the Res2Net module is introduced into the backbone feature extraction network to improve the feature extraction capability of the network. In addition, a detection scale is added to the detection part of the network to expand the sensing domain of the network. Finally, the average detection accuracy of the algorithm reaches 80.1%, with detection speed reaching 76.9 frames/s. Yang and Sang (2022) uses the improved YOLOv4 algorithm to detect fabric flaws. It uses the lightweight network MobileNetv2 as the main feature extraction network of YOLOv4 and adds the CA mechanism into the inverse residual structure of MobileNetv2 to improve the feature extraction ability of the model for small targets. In addition, the adaptive spatial feature fusion (ASFF) structure is used to improve PANet, so that the model can obtain the fusion weight of multi-scale feature map through learning so that the shallow feature and deep feature can be fully utilized, and the accuracy of small target flaw detection can be further improved. Finally, the average detection accuracy of the algorithm reaches 91.67%, with detection speed reaching 26 frames/s.

As can be seen from the flaw detection results of white porcelain bottles by various methods in Table 1, as a popular traditional method in the two-stage field, Faster-RCNN has higher accuracy than YOLOv3, but lower detection speed. YOLOv3 is not only low accuracy but also slow speed which is not suitable for the real-time detection of white porcelain bottles in industrial scenes. YOLOv4 is ahead of Faster-RCNN and YOLOv3 in mAP and FPS, and its detection speed and accuracy still have room for improvement. The detection speed of YOLOv5 is faster than that of YOLOv4, but the detection accuracy is lower than that of YOLOv4. The algorithm in this research is improved on the basis of YOLOv4. Compared with YOLOv4, the improved algorithm improves mAP by 5.2%. Although the detection speed is reduced by 1.33 frames/s, it still meets the real-time detection requirements of white porcelain bottles.

By comparing the experimental results in this research with Qiu et al. (2022), it can be seen that the average detection accuracy of the algorithm is 12.46% higher than that in Qiu et al. (2022), but the detection speed of the algorithm is 39.73 frames/s lower than that in Qiu et al. (2022). This is because Qiu et al. (2022) uses the YOLOV4-tiny algorithm, a simplified version of the YOLOv4 algorithm, for flaw detection. The YOLOV4-tiny algorithm is a lightweight algorithm with fast detection speed but relatively low detection accuracy. By comparing the experimental results in this research with Yang and Sang (2022), it can be seen that the improved algorithmis superior to Yang and Sang (2022) in both average detection accuracy and detection speed. Yang and Sang (2022) In order to improve the detection speed of the algorithm, the trunk network of YOLOv4 was replaced with MobileNetv2, but the average detection accuracy of the algorithm decreased. Then, Yang and Sang (2022) improved the average detection accuracy of the algorithm by adding CA mechanism to the trunk feature extraction network and improving the PANet structure, but it also brought a certain amount of calculation, resulting in a decrease in the detection speed of the algorithm. However, the proposed algorithm only adds CA mechanism and deformable convolution to the backbone feature extraction network of YOLOv4, which improves the detection accuracy of the network and also introduces a certain amount of computation, resulting in a decrease in detection speed. After that, the loss function was improved to improve the detection accuracy of the algorithm and meet the real-time requirements of white porcelain wine bottle flaw detection in the industrial setting.

Through comparative experimental data analysis, the proposed algorithm can achieve higher detection accuracy and faster detection speed in white porcelain wine bottle flaw detection, which is suitable for the real-time detection of white porcelain wine bottles in the industrial setting.

### Ablation Experiments

In order to verify the effects of CA mechanism, deformable convolution, and EIoU Loss function on model performance, ablation experiments were performed on the added modules using the YOLOv4 algorithm. The impact of different modules on model performance is shown in Table 2.

**TABLE 2:** Effects of different modules on model performance.

Model	CA	DCN	EIoU	AP/%			mAP/%	FPS/(frames/s)
				Gap	Crack	Stain		
YOLOv4	—	—	—	89.15	86.71	86.22	87.36	38.5
YOLOv4_1	✓	—	—	92.32	88.58	89.61	90.17	34.7
YOLOv4_2	—	✓		91.13	90.18	87.37	89.56	36.5
YOLOv4_3	—	——	✓	89.93	87.35	86.9	88.06	41.97
YOLOv4_4	✓	✓	—	93.69	90.78	90.54	91.67	33.2
Improved YOLOv4	✓	✓	✓	94.75	91.56	91.37	92.56	37.17

By analyzing various data in Table 2, it can be concluded that the mAP value increased from 87.36 to 90.17% after the CA mechanism was added to the YOLOv4 trunk feature extraction network, an increase of 2.81%. The detection accuracy of notches and stains improved greatly, and their AP value increased by 3.17 and 3.39%, respectively, while the AP value of cracks increased by 1.87%. This is because in the white porcelain wine bottle data set, there are more small targets for the two flaws of gap and stain, while there are fewer small targets for the flaws of crack. The CA attention mechanism can improve the network’s attention to feature information, so that the model can pay attention to more small target feature information. Therefore, the addition of CA attention mechanism can greatly improve the detection accuracy of gap and stain, while the detection accuracy of crack is relatively small. After the CA mechanism is added, the feature extraction ability of the YOLOv4 backbone network is improved, so the detection accuracy of the model is improved. However, the calculation amount is increased, so the detection speed of the model is slightly decreased. After deformable convolution (DCN) was added to the backbone feature extraction network of YOLOv4, the mAP value increased from 87.36 to 89.56%, an increase of 2.2%. The AP value of cracks increased by 3.47%, and the AP value of notches and stains increased by 1.98 and 1.15%, respectively. This is because there are various shapes and sizes of flaws such as cracks in the white porcelain wine bottle data set, and deformable convolution (DCN) can dynamically adjust the flaws of different shapes and sizes, thus improving the detection accuracy of the model. At the same time, the addition of deformable convolution also brings a certain amount of computation, which makes the detection speed of the model decrease slightly. After replacing the loss function CIoU of YOLOv4 with EIoU, the mAP value increased slightly, only increasing by 0.7%, but the detection speed of the model increased from 38.5 to 41.97 frames/s, 3.47 frames/s. After the CA module, deform convolution (DCN) and EIoU were added into YOLOv4, the detection accuracy was greatly improved, and the mAP value increased from 87.36 to 92.56%, an increase of 5.2%. Although the detection speed is reduced by 1.33 frames/s compared with YOLOv4, it still meets the real-time requirements of white porcelain wine bottle flaw detection in the industrial setting.

### Detection Results and Analysis

**FIGURE 9:**
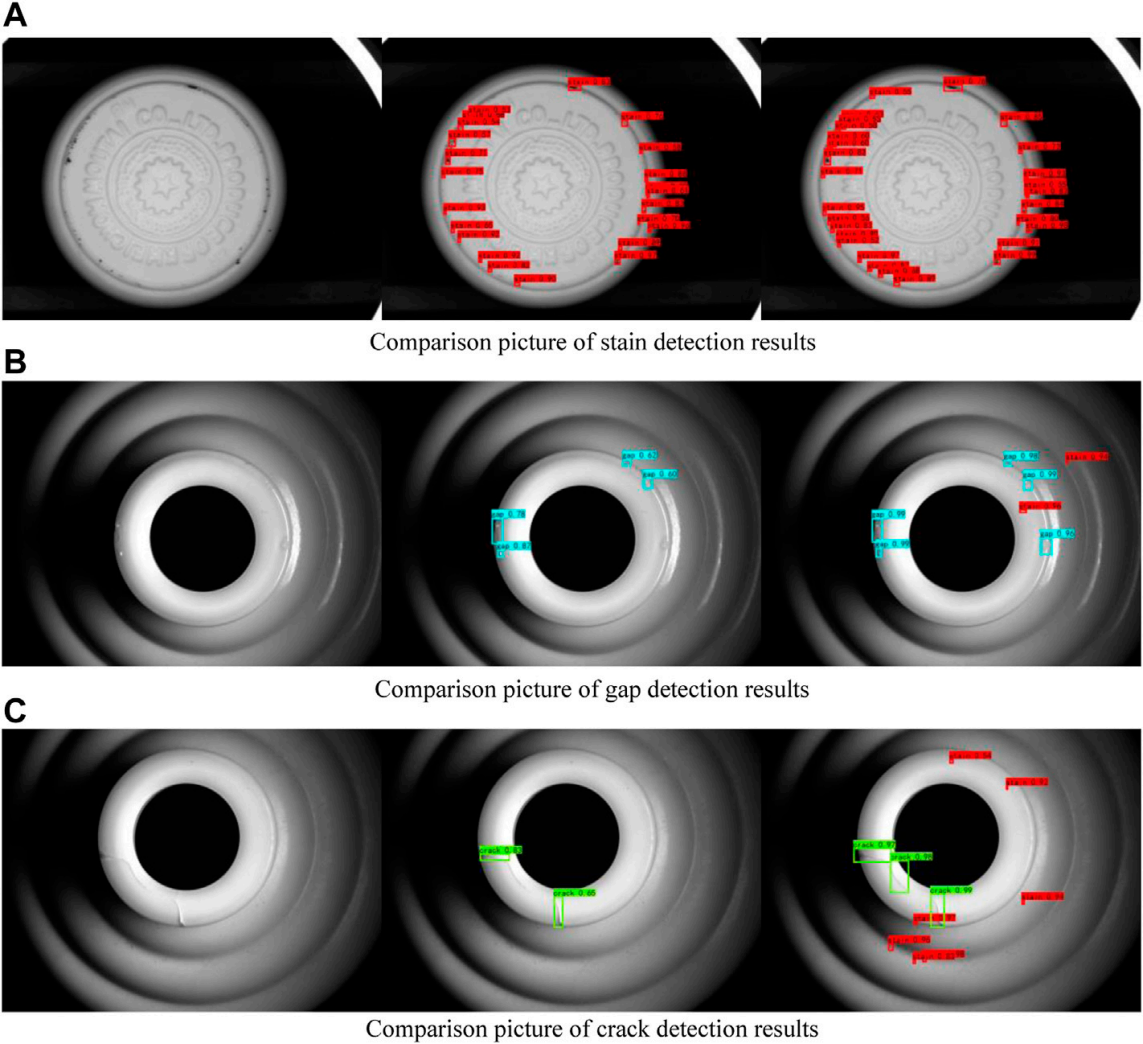
Comparison of detection results of different flaws between this algorithm and YOLOv4. **(A)** is the contrast diagram of the gap detection results of white porcelain bottle, **(B)** is the contrast diagram of the crack detection results of white porcelain bottle, **(C)** is the contrast diagram of the stain detection results of white porcelain bottle.

In order to better verify the feasibility of the improved algorithm presented in this research, white porcelain wine bottles with different flaws were selected for testing in the test set. Figure 9 shows the comparison of detection results of different flaws between the improved YOLOv4 algorithm and the original YOLOv4 algorithm in this research.

Among the three groups of detection results in Figure 9, the leftmost image is the original image, the middle image is the detection result of YOLOv4 algorithm, and the rightmost image is the detection result of the three kinds of flaws by the improved YOLOv4 algorithm in this research. As can be seen from the comparison figure of stain detection results, YOLOv4 can detect some large stain flaws in the images, but it cannot detect small target stains in the images. However, the improved algorithm in this paper can detect small stain flaws due to the addition of CA attention mechanism, which improves the attention of the network to small targets. As can be seen from the comparison figure of gap detection results, due to uneven illumination, part of the gap area has a high brightness, which makes the gap similar to the background of the white porcelain bottle. YOLOv4 can detect a relatively obvious gap, but there is the issue of missing detection for some obscure gaps. In this research, the improved algorithm introduces the CA mechanism, which improves the attention of the network to the flaw target and reduces the attention of the network to the background target, so that the network can detect the flaw target more accurately. As can be seen from the comparison figure of crack detection results, for cracks of different shapes and sizes, YOLOv4 has missed detection. However, due to the addition of deformable convolution to the improved algorithm in this research, the network can more accurately locate cracks of different shapes and sizes, thus improving the accuracy of crack detection. As can be seen from the comparison figure of the experimental results of the three groups of flaw detection, the improved YOLOv4 algorithm in this research greatly improves the missed detection of the original YOLOv4 and improves the confidence of flaw detection. Therefore, it can be shown that the CA mechanism increases the model’s attention to small targets and fuzzy targets. Deformable convolution improves the model’s attention to flaws of different shapes and sizes, both of which greatly improve the detection accuracy of the network for flaw targets.

## Conclusion

This research presents an improved YOLOv4 algorithm for flaw detection in white porcelain wine bottles. The CA attention module is added to the backbone feature extraction network of YOLOv4, this improves the detection accuracy of the model for small target flaws. On this basis, some ordinary convolutions in the backbone feature extraction network are replaced with deformable convolutions, so that the model can better locate flaws with different shapes and sizes, and further improve the detection accuracy of the model. Finally, the loss function CIoU of YOLOv4 was replaced by EIoU to accelerate the convergence of the model, improve the detection accuracy and speed. Experimental results show that in the white porcelain wine bottle flaw detection task, the average detection accuracy mAP of the proposed algorithm can reach 92.56%, which is 5.2% higher than the original YOLOv4 algorithm. The detection speed of the proposed algorithm reaches 37.17 frames/s, which meets the real-time requirements of the white porcelain wine bottle flaw detection in the industrial setting. However, the algorithm presented in this research can be further improved, specifically with regard to detection speed and detection accuracy can be further improved. In the future, in solving the problem of detection speed, on the basis of ensuring detection accuracy, the main network of the model will be changed to reduce the size of the model, reduce the amount of calculation, and further improve the detection speed of the model.

## Data Availability

The raw data supporting the conclusion of this article will be made available by the authors, without undue reservation.
